# Fine‐scale variation within urban landscapes affects marking patterns and gastrointestinal parasite diversity in red foxes

**DOI:** 10.1002/ece3.6970

**Published:** 2020-11-19

**Authors:** Lisa V. Gecchele, Amy B. Pedersen, Matthew Bell

**Affiliations:** ^1^ Ashworth Laboratories School of Biological Sciences Institute of Evolutionary Biology University of Edinburgh Edinburgh UK

**Keywords:** gastrointestinal parasites, landscape fragmentation, urban carnivores, urban disease ecology, urban ecology, *Vulpes vulpes*

## Abstract

1. Urban areas are often considered to be a hostile environment for wildlife as they are highly fragmented and frequently disturbed. However, these same habitats can contain abundant resources, while lacking many common competitors and predators. The urban environment can have a direct impact on the species living there but can also have indirect effects on their parasites and pathogens. To date, relatively few studies have measured how fine‐scale spatial heterogeneity within urban landscapes can affect parasite transmission and persistence.
2. Here, we surveyed 237 greenspaces across the urban environment of Edinburgh (UK) to investigate how fine‐scale variation in socio‐economic and ecological variables can affect red fox (*Vulpes vulpes*) marking behavior, gastrointestinal (GI) parasite prevalence, and parasite community diversity.
3. We found that the presence and abundance of red fox fecal markings were nonuniformly distributed across greenspaces and instead were dependent on the ecological characteristics of a site. Specifically, common foraging areas were left largely unmarked, which indicates that suitable resting and denning sites may be limiting factor in urban environments. In addition, the amount of greenspace around each site was positively correlated with overall GI parasite prevalence, species richness, and diversity, highlighting the importance of greenspace (a commonly used measure of landscape connectivity) in determining the composition of the parasite community in urban areas.
4. Our results suggest that fine‐scale variation within urban environments can be important for understanding the ecology of infectious diseases in urban wildlife and could have wider implication for the management of urban carnivores.

## Graphical abstract

We surveyed 237 greenspaces across the urban environment of Edinburgh (UK) to investigate how fine‐scale variation in socio‐economic and ecological variables can affect red fox (*Vulpes vulpes*) marking behaviour, gastrointestinal (GI) parasite prevalence, and parasite community diversity. We found that the presence and abundance of red fox faecal markings were nonuniformly distributed across greenspaces and instead were dependent on the ecological characteristics of a site. Specifically, common foraging areas were left largely unmarked, which indicates that suitable resting and denning sites may be limiting factor in urban environments. In addition, the amount of greenspace around each site was positively correlated with overall GI parasite prevalence, species richness, and diversity, highlighting the importance of greenspace (a commonly used measure of landscape connectivity) in determining the composition of the parasite community in urban areas.

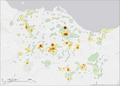

## 1 INTRODUCTION

Urban areas are expanding around the world due to both the increase in overall human population size and the trend of migration from the countryside to larger settlements (United Nations, 2008). These urbanized areas can represent “virgin” ecosystems, as they often are devoid of resident species, which can be a challenging environment for wildlife (Lowry et al., 2013; McIntre et al., 2000). In addition, urban habitats are highly disturbed and can be very fragmented (Fernandez‐Juricic, 2000), due to traffic (Magle et al., 2009), noise pollution (Francis et al., 2011), light pollution (Miller, 2006), and the presence of people (Schlesinger et al., 2008). While these conditions may provide challenges for most wildlife species, urban areas also tend to have very abundant and stable food sources (McKinney, 2006) and fewer predators and competitors than wild habitats (Crooks & Soulé, 1999). Due to these unique ecological conditions, relatively few species have successfully adapted to exist, and in many cases thrive, in urban environments (Lowry et al., 2013), with the classic examples of pigeons and rats (Luniak, 2004).

Ecological species assemblages, or communities, that exist in urban environments tend to have a different composition from those found in wild settings (Aronson et al., 2016), but they are not necessarily less diverse (Parsons et al., 2018). Urban communities often consist of species capable of tolerating highly disturbed habitats and able to exploit novel resources (Luniak, 2004). While there is an increasing focus on understanding the ecological communities that emerge in urban areas, the consequences for how the urban landscape may affect the parasite community remains unclear (Bradley & Altizer, 2006). For this reason, understanding the factors driving parasite diversity, infection risk, and parasite burdens in urban wildlife can be important to unravel the dynamics of transmission at the wildlife–human and urban–wild interfaces (Gortázar et al., 2007). For example, fragmented landscape and the associated decrease in biodiversity found in urban areas are correlated with an increase in the density of white‐footed mice (*Peromyscus leucopus*), hosts for the spirochete bacteria (*Borrelia burgdorferi*) that cause Lyme disease. Moreover, urbanization can provide suitable habitat to some species that would not normally live in close proximity with humans, increasing the risk of cross‐species or zoonotic transmission (Hassell et al., 2017). For example, the presence of flying foxes (*Pteropus*) in urban areas has been associated with the spillovers of Hendra virus to humans and domestic animals in Eastern Australia (Plowright et al., 2011).

Recently, there has increased effort to identify more generalizable patterns about the impact of urbanization on the structure and dynamics of parasite communities (see, e.g., Murray et al., 2019; Werner & Nunn, 2020). However, these studies have focused on comparing urban to rural landscapes, without taking into count the enormous variation in the physical and biological composition of urban areas, which leads to highly variable urban ecological communities (McKinney, 2006). Therefore, patterns and drivers of parasite infection, burden, and diversity are unlikely to be consistent across urban areas (Bradley & Altizer, 2006). A clear example of this variation has been demonstrated through the in‐depth investigation of *Echinococcus multilocularis,* a tapeworm with a complex life cycle, which causes alveolar echinococcosis, a zoonotic disease of increasing importance for humans across Europe. Red foxes are a competent definitive host for *E. multilocularis*, and the presence of high‐density fox populations in urban areas has sparked concern for public health in endemic regions (Mackenstedt et al., 2014). In a review of *E. multilocularis* in urban fox populations, Deplazes et al. (2004) concluded that urban foxes consume a lower proportion of small rodents (the intermediate hosts), which leads to lower infection rate in urban foxes compared to rural settings. However, further work that included recent research from China and Japan found contrasting results; higher *E. multilocularis* prevalence was associated with more urbanized areas in rural China, a pattern that was driven by the presence of free‐roaming dogs, a key definitive host for *E. multilocularis* (Liccioli et al., 2015).

These contrasting patterns highlight the importance of taking into account the specific characteristics of urban landscapes in determining their impact on host–parasite dynamics. However, producing a comprehensive definition of urban areas that incorporate this intrinsic variability is difficult (Weeks, 2010). In fact, most studies use subjective classifications to identify specific environments within urban areas, generally defining urban versus rural areas (see, e.g., Fischer et al., 2005; Prange et al., 2003; Reperant et al., 2007; Robardet et al., 2008). However, “urban” and “rural” landscapes are just ends of a continuous spectrum of urbanization, and to understand the dynamics and structure of parasite communities in urban environments, it is important to be able to classify specific characteristics of urban areas or the level of “urbanity,” along this spectrum (McDonnell & Pickett, 1990). These metrics need to be objective and quantitative and capture the heterogeneity and fragmentation of the urban environment on a fine scale, while also being able to accurately characterize the rural–urban transition within continuous multivariate space.

Red foxes (*Vulpes vulpes*) are an extremely adaptable species (Harris & Baker, 2001), with a generalist diet (Contesse et al., 2004), high reproductive potential (Pagh et al., 2018), and a flexible social system (Iossa et al., 2008); these traits have allowed foxes to adapt to urban environments and quickly establish dense populations (Harris, 1981; Janko et al., 2012). Records from the early 1900s in London suggest that urban fox populations were already well established (Teagle, 1967). Patterns of long‐standing urban fox populations have been reported in numerous countries, particularly in Canada, Australia, Japan, and mainland Europe (Harris & Rayner, 1986; Adkins & Stott, 1998; Gloor et al., 2001; Marks & Bloomfield, 2006; Uraguchi et al., 2014). However, urban foxes have often been regarded as pests because they can carry important zoonotic diseases (e.g., rabies virus, *E. multilocularis*) which generate concern for public health (Comte et al., 2013; Laurimaa et al., 2016; Reperant et al., 2007).

Here, we developed a multivariate, continuous measure of “urbanity” in order to investigate the effect of fine‐scale habitat changes on the abundance of fox territorial marking and the composition of their gastrointestinal (GI) parasite communities across Edinburgh, UK. To do this, we conducted an extensive, noninvasive survey of public greenspaces across the entire urban area of Edinburgh, recorded all red fox scats to identify fox distribution patterns, and identified and quantified the GI parasite community. We used fine spatial‐scale metrics that included both human socio‐economic variables (i.e., human population density, traffic counts, and greenspace) and ecological variables (i.e., the presence of other wildlife species and habitat characteristics), to capture the complex biotic and abiotic structure of the urban environment and investigate their relationship with parasite diversity and infection prevalence. Our goal was to identify variables drive patterns of GI parasite infection in the urban landscape, in order to provide an objective and easily quantifiable measure of urbanity, as to improve comparability and repeatability of urban disease ecology studies.

## 2 METHODS

### 2.1 Study area and survey design

**FIGURE 1:**
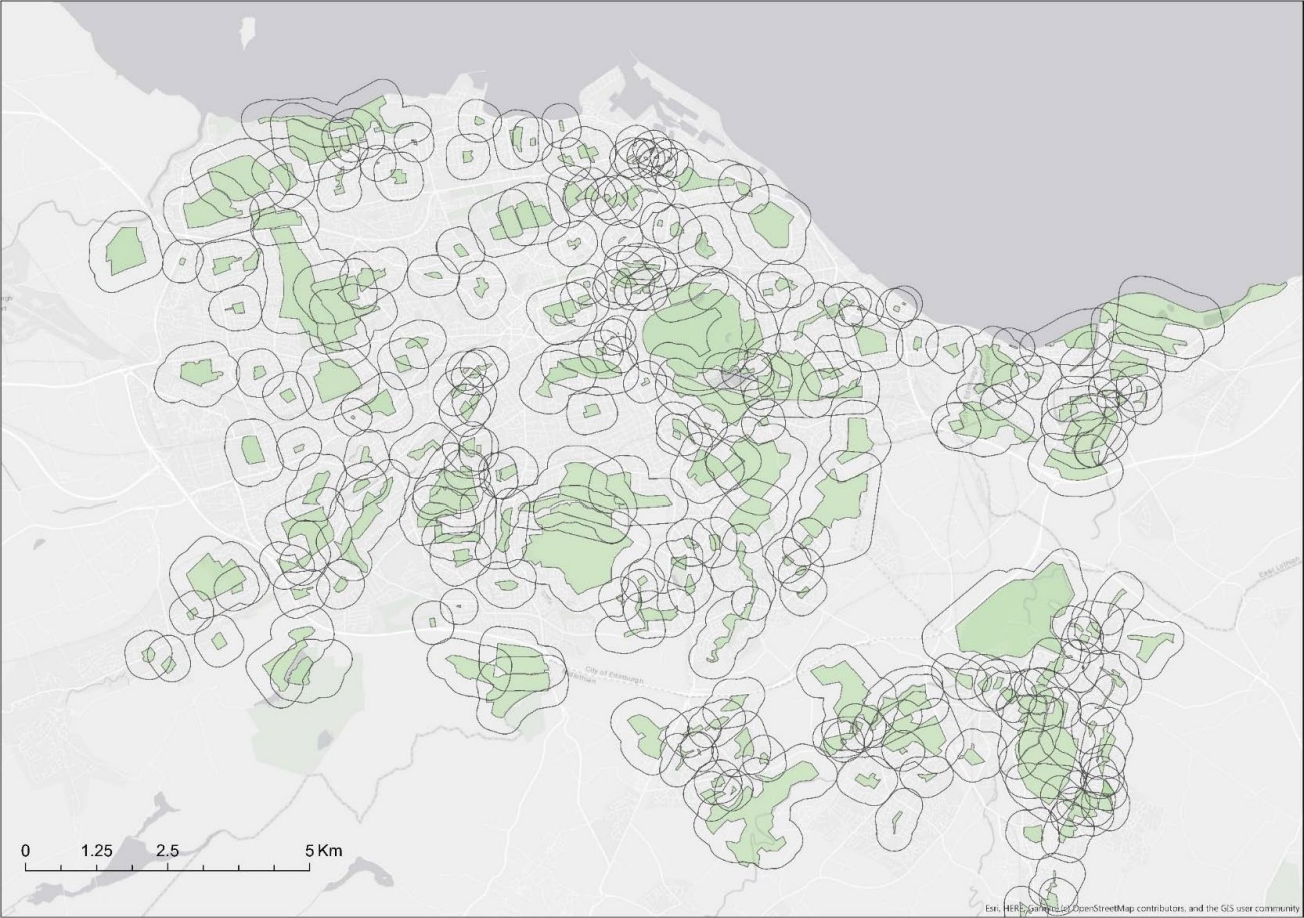
Map of Edinburgh detailing the 273 greenspace survey sites (in green) with a 300 m planar radius buffer used to calculate the urbanity measures. All sites were sampled during both of the two sampling periods (spring and autumn) in 2017

Fieldwork was carried out in the urban area of Edinburgh, United Kingdom (55.9533°N, 3.1883°W). We identified study sites using the greenspace database (http://digimap.edina.ac.uk/os), specifically, we selected all areas classified as public greenspaces (i.e., public parks, playgrounds, golf courses, and natural areas) within the city limits. In total, this included 329 unique sites, varying in size between 0.0002 and 1.684 km^2^; with an average site area of 0.135 ± 0.22 km^2^. Of this set of greenspaces, we were able to survey 273 unique sites; as 56 sites (17%) were not accessible, had been repurposed, or no longer existed. The total extent of the urban Edinburgh study area was 213.35 km^2^, and the surveyed sites covered 16.7% of the total area (Figure 1).

We surveyed each site twice in 2017. The two sampling periods were chosen to capture differences in red fox life history i) the “Spring” season (25th January to 4th May 2017) coincided with the period between breeding and cub emergence from the den; ii) the “Autumn” season (1st August to 5th October 2017), captured cub weaning and preceded the dispersal of subadults (Hewson & Kolb, 1980; Harris, 1981). At each site for each sampling period, we surveyed the perimeter of the greenspace and recorded the presence of all fox scats (fecal samples). Morphological identification of the scats was based on content (e.g., whether it contained bone fragments, hair, or feathers), shape, size, and color (Heinemeyer et al., 2008). For each scat, we recorded GPS coordinates and if it was freshly deposited (i.e., not moldy and still dark in color), the sample was collected, weighed, and stored in 10% buffered formalin solution at 4**°**C for further analysis.

### 2.2 Socio‐economic and ecological variables

**FIGURE 2:**
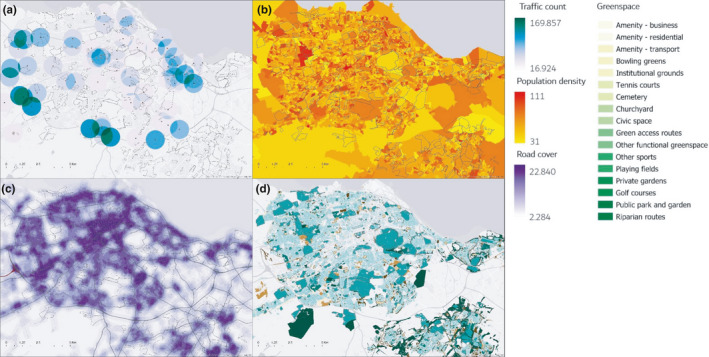
Maps of Edinburgh (UK), showing the range and distribution of the four socio‐economic datasets used in the analyses. (a) Traffic counts, expressed in average number of vehicles per day. (b) Population density, expressed in number of people per hectare. (c) Road cover, expressed in Km of road per km. (d) Greenspace, mapped according to the classification of the original dataset (OS open greenspace). All four datasets are mapped using a 30 × 30 m raster, and the data relative to each site (plotted in gray in each map) surveyed are extracted as average of pixel value within a 300 m planar buffer area around each site (see Figure 1)

In order to effectively capture fine‐scale variation in the urban landscape, we measured both socio‐economic and ecological variables. First, we identified several socio‐economic variables that reflect aspects of anthropogenic disturbance that are typically representative of urbanity (Hahs & McDonnell, 2006): human population density, road cover and traffic counts, and the ratio and variability of greenspace (Figure 2). We collated data for each site from publicly available databases:

- Resident human population density *(* http://www.scotlandscensus.gov.uk ) is widely used as a proxy for urbanity and broadly reflects human abundance and land use (du Toit & Cillier, 2011).
- Road cover (http://digimap.edina.ac.uk/os) and traffic counts (http://www.dft.gov.uk/traffic‐counts). Roads can act as barriers to dispersal (Magle et al., 2009), by altering the geophysical characteristics of the environment (Gaston et al., 2010; Yuan & Bauer, 2007) and their distribution correlates with habitat disturbance (Arnold & Gibbons, 1996). Traffic is the leading cause of fox mortality in cities (Gosselink et al., 2007) which can generate marked changes in the demographic structure of urban fox populations (Baker et al., 2007).
- Greenspace ratio and variability (http://digimap.edina.ac.uk/os). Greenspaces are the most important urban areas as they provide suitable sites for wildlife to rest and breed. Greenspaces are defined as urban green areas such as parks and sports facilities, where building is limited or absent and where some form of vegetation is the primary land cover; (Taylor & Hochuli, 2017). Greenspaces are vital for urban foxes (Baker et al., 2007), which are primarily active during the night and require safe hiding spots to rest during the day (Harris & Baker, 2001). In particular, areas of continuous suitable habitat play an important role in the connectivity of the urban landscape and can allow foxes to move around the urban areas relatively undisturbed (Schiller & Horn, 1997).

We also recorded the following four ecological variables that describe important aspects of the biotic habitat for each greenspace site surveyed:

- The presence/absence of European rabbits (*Oryctolagus cuniculus*). These medium‐sized (2–4 kg) lagomorphs are one of the main prey species of red foxes (Lees & Bell, 2008) and are present across Edinburgh.
- The presence/absence of roe deer (*Capreolus capreolus*). The presence of large ungulates, such as roe deer, while not directly related to fox diet as they are too large to be prey, serve as a useful proxy indicator for the overall “wilderness” of a site (Magle et al., 2014).
- The presence/absence of European gorse (*Ulex europaeus*). The vegetation of this plant, which is found across both urban and rural habitats across the UK, is particularly impenetrable. It can create secure, suitable microhabitats within human‐dominated or disturbed greenspaces where foxes, and other urban wildlife, may safely rest and breed (White et al., 1996).
- Vegetation management regime. Management of greenspaces can vary from high‐intensity amenity grassland to very low‐intensity seminatural woodland or moorland, with likely consequences for prey abundance and diversity (Goddard et al., 2010), the availability of resting and denning sites, and the extent of human incursion and disturbance. We assessed this index as the intensity of the management rather than its extent, on a scale from 0 (the site vegetation was left completely untouched), to 4 (large portions of the site were actively managed throughout the year; e.g., by cutting the grass).

**FIGURE 3:**
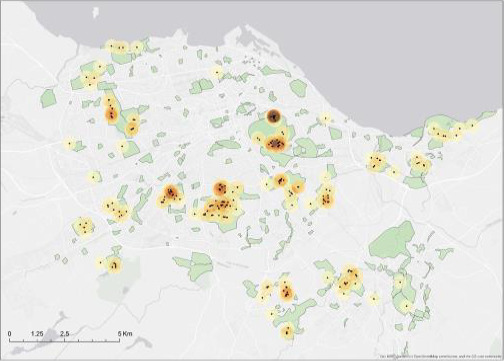
Map of the study area (urban Edinburgh), highlighting all the survey sites in green (*n* = 273; 16.7% of the entire map area). All red fox scats that were found across both seasons are represented by black dots. The density of scats across the area is mapped using a kernel density function with a radius of 300 m. The color represents the number of scats found per square meter from dark orange to transparent (min = 0.00024, max = 0.0255)

Each socio‐economic variable defined above was mapped across the entire study area using a 25 × 25 m raster ArcGIS pro 1.4 (ESRI, 2017). We extracted the average value relative to each site using the zonal statistics tool. For the greenspace variability metric, we obtained both the average greenspace cover (i.e., ratio of green area/total area) and the variability of greenspace, which is expressed as the number of different greenspace categories (out of the 25 identified by remote sensing in the dataset) (Figure 3).

**FIGURE 4:**
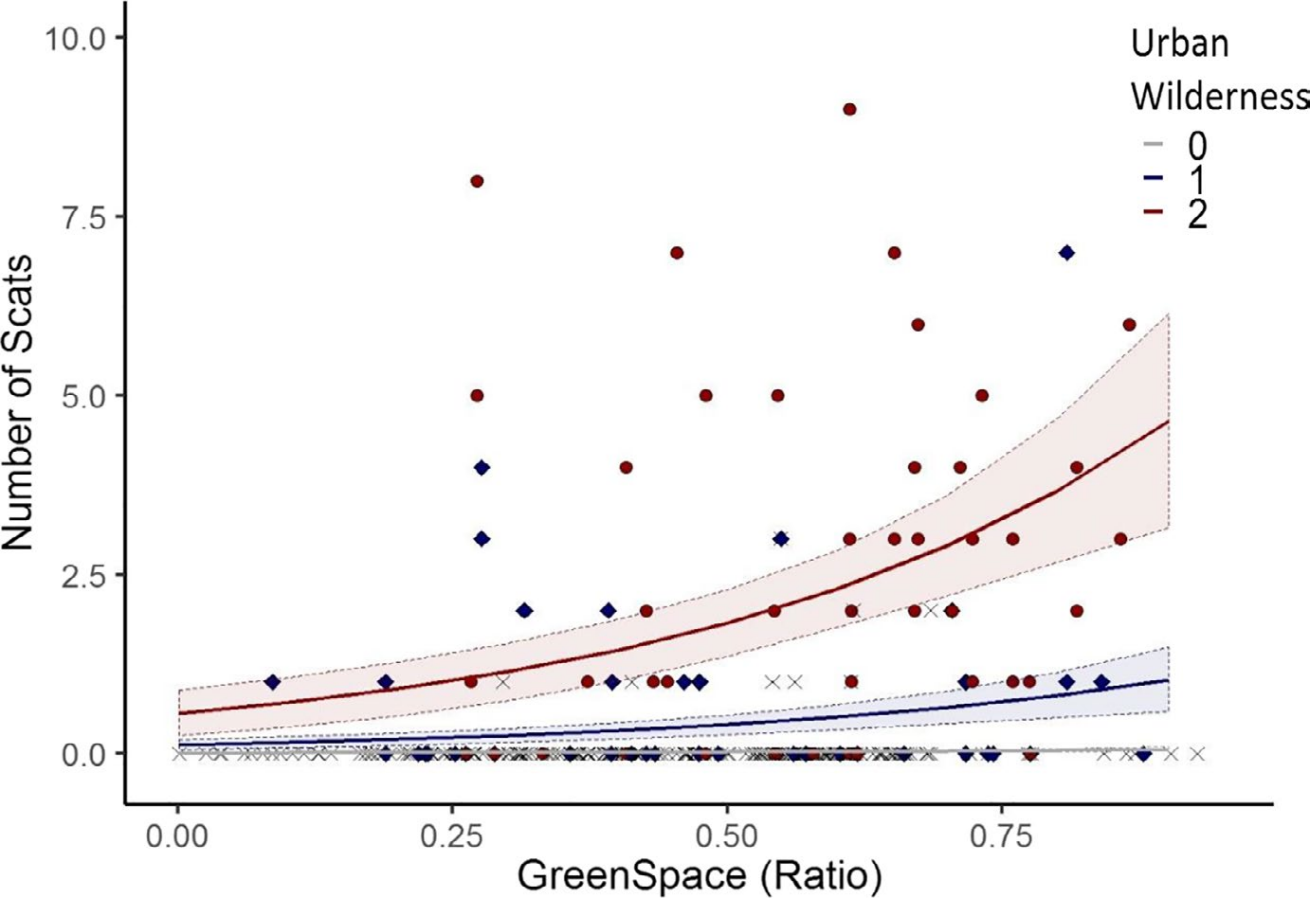
Model prediction from the GLMM on number of red fox scats found per site. Greenspace ratio was positively associated with the number of scats found; we also found a positive relationship with “urban wildness” score, with a higher number of fox scats found in sites where we record the presence of other species (roe deer, rabbits, and gorse). The gray line represents greenspaces with no wild species found, the blue line represents sites with one species, and the red one sites with two or three species recorded. The raw data are also included in the figure as specific points, in the same color scheme

Given previous estimates of urban fox home ranges (~1.75 km^2^ for resident adults; Castañeda et al., 2019), it is likely that foxes living in a specific greenspace will roam to neighboring areas and could be affected by the level of urbanity beyond the specific sites where scats were found. To account for this, we extracted the values for each socio‐economic variable from the surrounding areas, by including a buffer polygon with a radius of 300 planar meters around each site, which was chosen to reflect the reported average distance travelled by foxes in nondispersing movements (Iossa et al., 2008). Each buffer area included landscape features that were most likely to represent the habitual home range of the foxes living in each site. All socio‐economic variables were continuous but measured in different scales, thus we standardized each to a mean of zero and variance 1 to avoid convergence problems in the models (Figure 4).

### 2.3 Gastrointestinal (GI) parasite community

All fresh fox fecal samples were analysed using salt flotation and microscopy in order to identify parasite species and abundance, based on egg/oocyst morphology using a Veterinary Parasitology key (Foreyt, 2001). When possible, we identified each parasite to species level, however, in some cases this was not possible, and so, we identified each parasite to the lowest taxonomic level possible. For each fox fecal sample, we recorded: (a) the presence/absence (0/1), (b) burden (eggs/oocysts per grams) for each parasite taxon, (c) species richness (number of GI parasite species/taxon), and the (d) Shannon diversity index. Specifically, the Shannon diversity index (H) was calculated using the package vegan (Oksanen et al., 2015). This is a metric commonly used to evaluate the diversity of an assemblage, taking into account both relative abundance and species richness (Chao et al., 2014).

### 2.4 Statistical analysis

To determine whether there was a pattern in the distribution of red fox scats found across greenspaces in urban Edinburgh, we fit a generalized linear mixed model (GLMM) with the socio‐economic and ecological variables (details below) as predictors of red fox marking (number of scats found per site). Next, we fit a series of GLMMs to determine the impact of urbanity on the fox parasite community. Specifically, we tested the effect of the socio‐economic and ecological variables on: (a) overall parasite infection risk (the presence/absence of each parasite species), (b) parasite species richness, and (c) parasite community composition (Shannon diversity index). Finally, we fit individual models to test the effect of these variables on single parasite species infection risk (presence/absence) and burden (eggs/oocyst per gram).

We also tested for spatial autocorrelation in the dataset using a variogram of Pearson's residuals from each model (fitted without a spatial term, see Supplemental material). All models showed evidence of spatial autocorrelation, and so, we included a spatial term using a conditional autoregressive correlation model with a Matérn correlation structure in each model (Rousset & Ferdy, 2014) using the package spaMM (Rousset et al., 2018). All models were fit using the function HLfit (adjusted by maximum likelihood), and the fixed effects were tested for significance using the function fixed LRT.

Before fitting each model, we also checked for collinearity between the variables by calculating the variance inflation factor, which provides an index measuring how much the variance of the estimated regression coefficient is increased because of collinearity. we used a value of 2 as cutoff for exclusion (following Craney & Surles, 2002), which revealed that some of the ecological variables which were fit as factors (specifically the presence/absence of rabbits, roe deer, and gorse) were significantly correlated. Therefore we combined these three ecological metrics into a single composite variable called “urban wilderness” which had three levels (“0,” “1,” and “2 or 3”), representing the number of these three possible species recorded at each site.

Each model included the following fixed effects measured at each site (inclusive of the 300 m buffer around each greenspace): five socio‐economic variables (population density, road cover, traffic counts, greenspace ratio, and variability) and two ecological variables (urban wilderness and the level of vegetation management). We also included the sampling period as a factor (spring and autumn), and site area (m^2^; log‐transformed) and site transect length (m) as an offset to account for the different transect lengths in each site. The first model which tested how these variables impacted the number of scats found per site was fit using a Poisson distribution and included Site ID and a Matérn spatial correlation structure as random effects. To investigate the drivers of parasite species richness (count data) and the Shannon diversity index (H; continuous), we fit Poisson and Gaussian distributions, respectively, and included Sample ID (species richness only), Site ID, and Matérn spatial correlation structure as random effects. Finally, the model fit to evaluate the likelihood of infection with all gastrointestinal parasite taxa (measured as the presence/absence per parasite taxon per sample), was fit using a Bernoulli distribution and included parasite taxon identity as a fixed effect and Sample ID, Site ID, and Matérn spatial correlation structure as random effects. The species‐specific models included a binomial (Bernoulli) distribution model fit on parasite prevalence (presence–absence of each parasite taxon per sample) and a Poisson distribution model fit on burden data (eggs/oocysts per gram).

## 3 RESULTS

### 3.1 Red fox scat distribution across urban Edinburgh

We sampled 237 greenspace sites across the urban environment of Edinburgh during two sampling periods in 2017, we found a total of 287 fox scats: 144 in spring and 143 in autumn (Figure 3). Overall, 224 (78%) were collected for gastrointestinal parasite analysis: 118 and 106, respectively, in spring and autumn.

Red fox scats were found only in 50 of the greenspace sites (18.3%), and this pattern was consistent across both seasons (35 sites in the spring, 36 in the autumn of which 21 had scats in both seasons). Furthermore, the average number of scats per marked site was 4.04 ± 2.40 (Range = 1–27; spring = 4.11 ± 3.82; autumn = 3.97 ± 5.24), indicating that if a site was marked, it was likely to contain multiple scats.

Importantly, we found the number of fox scats found at a site was determined by both socio‐economic and ecological factors. Specifically, both the greenspace ratio (*t* = 2.4432, *p* = 0.0233) and the composite “urban wildness” score (*t* = 4.0447 and *t* = 6.2009, respectively, for the two levels, *p* < 0.0001; Table 1) were significantly and positively associated with a higher number of scats found (Figure 4).

**TABLE 1:** The output for the model fit to determine which factors influence the number of scats found in each greenspace across urban Edinburgh

Fixed effects	Estimate	*SE*	*t*‐value	*p*‐value
Intercept	−12.029	1.885	−6.381	
Road cover	0.041	0.222	0.182	
Traffic counts	−0.017	0.149	−0.118	
Population density	−0.028	0.162	−0.174	
**Greenspace ratio**	**2.374**	**0.971**	**2.443**	*
Greenspace variability	0.021	0.0259	0.821	
Sampling period (autumn)	−0.373	0.212	−1.755	
Site area (Log)	0.052	0.159	0.329	
**Urban wilderness (1)**	**2.050**	**0.506**	**4.044**	***
**Urban wilderness (2–3)**	**3.166**	**0.510**	**6.200**	***
Veg management level (1–2)	−0.090	0.478	−0.189	
Veg management level (3–4)	−0.570	0.433	−1.316	

Random effects	Variance
Site ID	0.392
Spatial effect	0.355

Note

The variables included in the models are listed with model estimates, standard error (*SE*), and *t*‐values. The *p*‐values were calculated using a likelihood ratio test using the fixedLRT function in SpaMM. Highlighted the significant results in bold

* *p* < 0.005,

** *p* < 0.001,

*** *p* < 0.0001.

### 3.2 Gastrointestinal parasite community

We identified six parasite taxa from the fox fecal samples collected across Edinburgh. Specifically, we found four helminth taxa, including three nematodes and one cestode, and two species of coccidian parasites. Due to limitations with taxonomic resolution for parasite identification, we were able to identify three parasitic helminths to species level: *Toxocara canis, Eucoleus aerophilus, and Uncinaria stenocephala*, while the other three taxa were identified to genus level (helminth: *Taenia* spp. and coccidia: *Eimeria* spp. and *Isospora* spp).

Overall, 83.9% (188 out of 224) of the fecal samples contained helminth eggs or coccidian oocysts of at least one parasite taxon, and parasite infection (defined as infection with at least one taxon), was slightly higher in the spring (87.3%) than autumn (79.4%). There was also variation in the overall prevalence of each parasite taxon found, with helminth parasites being more common (79% of samples had at least one taxon) than coccidia. (45.9%; Table 2). The prevalence of different parasite taxa varied considerably, with only 8.9% of the samples found to contain *Taenia* 45% were positive for *U. stenocephala*. The average parasite species/taxon richness per sample was 1.96 ± 1.34 (spring = 2.18 ± 1.31; autumn = 1.72 ± 1.32; Table 2).

**TABLE 2:** Parasite prevalence (%) and average burden (average eggs/oocysts per gram of faeces, measured across all samples) for GI parasites of Red fox scat samples collected across greenspaces in urban Edinburgh in 2017, for each of the two survey seasons: Spring (January to April), and Autumn (August to October)

Taxon	Summer		Autumn	
	Prevalence	Burden	Prevalence	Burden
*Eimeria* spp.	42.8	24.59	30.8	199.57
*Isospora* spp.	10.1	0.24	17.7	2.08
**Total Coccidia**	**47.0**	**24.82**	**43.0**	**201.65**
*Eucoleus aerophilus*	52.1	1.08	32.7	3.13
*Toxocara canis*	14.3	2.68	13.1	14.41
*Uncinaria stenocephala*	47.1	4.59	42.0	4.70
*Taenia* spp.	12.6	1.10	4.67	0.12
**Total Helminth**	**82.3**	**11.32**	**72.9**	**11.82**

We found that the models fit to evaluate the effect of urbanization on the three broad GI parasite metrics (overall probability of infection, species richness, and parasite Shannon diversity) were qualitatively similar (Tables 3 and 4). All three analyses demonstrated a positive association between the parasite metric and greenspace ratio (*t* = 2.679, *p* = 0.007, *t* = 2.881, *p* = 0.008, and *t* = 3.238, *p* = 0.001 for infection probability, SR, and H index, respectively); while the second sampling period was negatively associated with three metrics (*t* = −2.419, *p* = 0.0215, *t* = −2.500, *p* = 0.022, and *t* = −2.350, *p* = 0.026 for infection probability, SR, and H index, respectively). Additionally, the Shannon diversity index of parasite diversity was found to be positively associated with both greenspace variability (*t* = 2.277, *p* = 0.0246) and traffic counts (*t* = 1.994, *p* = 0.0487).

**TABLE 3:** GLMM output of the Bernoulli model for the likelihood of gastrointestinal parasite species/taxa infection (presence–absence)

Fixed effects	Estimate	*SE*	*t*‐value	*p*‐value
*Eucoleus aerophilus*	1.246	2.296	0.542	
*Taenia* spp	−1.035	2.305	−0.449	
*Toxocara canis*	−0.508	2.300	−0.221	
*Uncinaria stenocephala*	1.330	2.296	0.579	
*Coccidian parasite*	1.351	2.296	0.588	
Road cover	0.117	0.138	0.851	
Traffic counts	1.477	0.162	0.109	
Population density	0.030	0.115	0.262	
**Greenspace ratio**	**2.671**	**0.996**	**2.679**	**
Greenspace variability	0.061	0.039	1.566	
**Sampling period (S2)**	**−0.478**	**0.198**	**−2.419**	*
Urban wilderness (1)	−0.006	0.524	−0.012	
Urban wilderness (2–3)	0.187	0.433	0.432	
Site area (Log)	−0.395	0.277	−1.424	
Veg. management level (1–2)	−0.274	0.437	−0.627	
Veg. management level (3–4)	−0.678	0.374	−1.811	

Random effects	Variance
Site ID	5.837e^−09^
Sample ID	0.1629
Spatial effect	0.3812

Note

The variables included in the models are listed on the left, along with estimate, *SE*, and *t*‐values. The *p*‐values for each fixed effect were computed applying a likelihood ratio test. Highlighted the significant results in bold

* *p* < 0.005,

** *p* < 0.001,

*** *p* < 0.0001.

**TABLE 4:** The GLMM model output for the GI parasite diversity (Shannon Index) and species richness models

Fixed effects	Species richness (SR)				Shannon diversity index (H)			
	Estimate	*SE*	*t*‐value	*p*‐value	Estimate	*SE*	*t*‐value	*p*‐value
Intercept	0.331	1.274	0.259		0.033	0.572	0.059	
Road cover	0.066	0.073	0.906		0.043	0.033	1.298	
Traffic counts	0.047	0.054	0.879		**0.053**	**0.027**	**1.994**	*
Population density	0.049	0.057	0.859		0.009	0.027	0.311	
**Greenspace ratio**	**1.511**	**0.524**	**2.881**	**	**0.779**	**0.238**	**3.268**	**
Greenspace variability	0.022	0.020	1.075		**0.021**	**0.009**	**2.277**	*
**Sampling period (autumn)**	**−0.263**	**0.105**	**−2.500**	*	**−0.118**	**0.05**	**−2.35**	*
Site area (Log)	0.189	0.302	0.627		−0.022	0.134	−0.169	
Urban wilderness (1)	0.208	0.249	0.837		0.005	0.112	0.044	
Urban wilderness (2–3)	−0.078	0.151	−0.515		−0.03	0.069	−0.42	
Managed vegetation level (1–2)	−0.253	0.232	−1.090		−0.11	0.108	−1.012	
Managed vegetation level (3–4)	**−0.417**	**0.188**	**−2.209**		−0.159	0.092	−1.719	
Random effects	Variance				Variance			
Site ID	6.58 e^−09^				4.67 e^−09^			
Spatial effect	9.55 e^−06^				6.43 e^−09^			
Sample					7.64 e^−09^			

Note

The variables included in the models are listed on the left, along with estimate, *SE* and *t*‐values. The variance explained by the random effects are also included at the bottom. Highlighted the significant results in bold

The *p*‐values for each fixed effect were computed applying a likelihood ratio test using the fixedLRT function in SpaMM.

* *p* < 0.05,

** *p* < 0.01,

*** *p* < 0.001.

The species‐specific models for the helminth parasite species included individual models fit for *E. aerophilus* and *U. stenocephala* prevalence and burden, *T. canis* prevalence. The models fit to *T. canis* and *Taenia* spp did not converge; likely d.ue to lack of data. Given the difficulty in identifying coccidian protozoans to the species level, we ran models evaluating the overall coccidian prevalence and burden (inclusive of both taxon). The models did not show any overall trend (see Appendix S1 for the full output of the models). We found that greenspace was positively correlated with *U. stenocephala* prevalence and burden (*t* = 2.037 and *t* = 2.238); *E. aerophilus* was less prevalent and less abundant in the second survey season (*t* = −3.199 and *t* = −4.177), and both *E. aerophilus* and the coccidian parasites prevalence were significantly correlated with the level of urban wilderness, but in opposite direction (*t* = −1.616 and *t* = 1.835, respectively). None of the variables included in the model had a significant effect on the prevalence of *T. canis*.

## 4 DISCUSSION

Using a comprehensive, repeated survey of greenspaces throughout the urban landscape of Edinburgh, we found evidence that fine‐scale landscape variation plays important roles in determining both red fox marking patterns and their gastrointestinal parasite infection community in Edinburgh. Specifically, we found that the amount of greenspace in and around each site (300‐m buffer) was positively associated with the number of fox scats found in a site (an indication of fox territorial marking) and GI parasite diversity, species richness, and likelihood of infection. Areas with a higher greenspace ratios contained more red fox scats, and in addition, these fecal samples were more likely to be infected with GI parasites, as well as having more diverse parasite communities.

Red fox scats had a distinct, highly localized, distribution across Edinburgh, and the strongest predictor for the number of scats found was the composite measure “urban wilderness.” This ecological metric included the number of other wildlife and plant species (specifically rabbits, gorse, and roe deer) present in a site. Specifically, sites that supported other wildlife/plants tended to be more heavily marked by red foxes, while sites with no or few of these species had far fewer red fox scats. In wild, nonurban habitats, red foxes have been shown to mark uniformly throughout their territory (Macdonald, 1980), and accordingly, we hypothesized that all the sites visited by foxes should be equally marked. However, our results suggest otherwise, and a clear pattern of marking emerged from our survey.

Most of the marked sites were considerably smaller than the reported size of urban fox home ranges (0.115–0.458 km^2^; Marks & Bloomfield, 2006), and therefore, it is unlikely that neighboring greenspaces would not be visited by resident foxes. Moreover, in a previous study of red fox movement conducted in the city of Edinburgh, Kolb (1985) reported extensive use of some habitat types that we found completely unmarked (e.g., cemeteries). In addition, our personal observational data support the hypothesis that red foxes can be found roaming and foraging on unmarked sites at night across Edinburgh (Gecchele et al., 2013). In addition, while we cannot exclude the fact that fox scats can be removed from certain greenspace areas and that this removal may vary between different green spaces, we believe that by measuring management of each site and including this in the models allowed us to control for this variation. It is likely that more intensely managed areas (e.g., cemeteries) would be more likely to remove red fox scats; however, we found no significant effect of management levels in our models. We suggest that a possible hypothesis for the observed nonuniform scat distribution patterns found across the urban landscape of Edinburgh is that scat marking is most concentrated in the core area of a foxes’ territories, while surrounding greenspace areas may be visited for foraging purposes, but are left unmarked. Greenspaces that host other wild species are more natural‐like and are more likely to provide suitable resting and denning sites; the higher number of scats found in these areas could be a result of higher territorial marking driven by competition for territories in these areas.

The ratio of greenspace within and around each site was the single most important predictor of GI parasite infection risk, species richness, and diversity, being positively correlated with all three metrics. Scats collected in sites surrounded by a higher amount of greenspaces were more likely to be infected with at least one parasite species and had a larger and more diverse parasite community. This metric of “greenspace ratio” can be considered a measure of landscape connectivity, since urban carnivores tend to use green areas to move around urban landscapes (Dodge & Kashian, 2013). As such, larger greenspace ratio scores suggest more contiguous greenspaces, which gives red foxes a chance to move safely across the urban environment (Kolb, 1984). At the same time, a higher concentration of scats in the marked sites may have important consequences for the transmission and infection burdens of gastrointestinal parasites of the foxes. Mathematical modeling has shown how increased marking rates can increase GI parasites’ prevalence (Nunn et al., 2011), while studies in raccoons suggest that the clustering of individuals can increase the prevalence and species richness of GI parasites (Wright & Gompper, 2005). A recent study conducted on red foxes from Berlin focused on the effect of the urban landscape structure on seroprevalence of canine distemper virus (CDV) using fox carcasses collected around the city. Similarly to our results, they found that amount of greenspace in the area surrounding the carcass was positively correlated with the probability of seropositivity, but only for juvenile animals (Gras et al., 2018). The authors concluded that access to more greenspace for juveniles was associated with a higher potential for dispersal and hence a higher risk of disease transmission. While we were not able to distinguish between individuals nor measure any demographic characteristics of the red foxes that deposited each scat, our results also suggest a connection between the amount of greenspace and the prevalence and diversity of GI parasites. This overall effect of greenspace ratio on parasite prevalence and abundance was not reflected on the single‐species models. These models did not show any broad trend, with several different variables having a significant effect on the prevalence and burden of different parasite species (including greenspace, sampling period, and urban wilderness). This could be due to reduced statistical power, as the data for a single parasite will be more zero‐inflated (in fact some models failed to converge).

Most previous studies of urban disease ecology have focused on how resource abundance and distribution in urban environment can affect host–parasite interactions (see, e.g., Becker et al., 2015; Bradley & Altizer, 2006; Mackenstedt et al., 2014). Here, we argue that we must move beyond a focus on resource availability; instead, we should be measuring fine‐scale variation in both socio‐economic and ecological factors that can determine how the urban landscape can impact parasites and pathogens. While food availability certainly plays a very important role in determining host–pathogens dynamics, it is generally really difficult to accurately measure the amount of anthropogenic food provided at a scale that is needed for most studies, while most variables used as proxy have proven not particularly accurate. For example, human density population has been shown to be positively correlated with the amount of anthropogenic food available to urban foxes in Zurich (Contesse et al., 2004), but this variable was not significantly correlated with either the presence of red fox scats, nor the GI parasite prevalence and diversity in our study. Instead, we find that the amount of greenspace and the presence of other “wild” species may be better indicators of how the habitat suitability varies across urban environments and may affect ecological interactions that lead to changes in the infection dynamics. Furthermore, given the increased importance of anthropogenic food sources purposely provided by residents which are closely associated with the presence of greenspace (e.g. pet food, birdfeeders, see), we believe greenspace ratio could potentially be a better proxy of anthropogenic food sources than previously used variables (such as human population density).

For logistic reasons, we only surveyed public greenspaces and were not able to collect data on fox scat distribution or GI parasite infection from private gardens/greenspaces. Previous studies on urban foxes have shown that private gardens can represent an important source of anthropogenic food for red foxes (Contesse et al., 2004), but the suitability of private urban gardens for resting and denning purposes has been debated. Saunders et al. (1997) found that back gardens were among the most favored habitat for day resting, while Newman et al. (2003) found that 86% of denning sites were located in back gardens. However, this dropped to 40% when population density crashed following a mange outbreak suggested that private gardens may not be the most desired nesting areas. Conversely, other studies of urban foxes found that back gardens, despite being the most used habitat, were not a suitable habitat for natal dens (Duduś et al., 2014). In addition, we lack direct information regarding each fox that deposited the scats and were unable to distinguish between individuals. However, we assume that fecal marking from a site is likely to be from a single fox group, given the territoriality of this species (Doncaster & Macdonald, 1991).

Another possible confounding factor in our analysis is the use of formalin as preserving medium used to store the samples. Storing fecal samples in formalin is common practice when flotation analysis cannot be performed immediately in order to preserve the parasite eggs as much as possible. While this can lead to a reduction of the identifiable eggs in the sample, this would be even in all our samples since they were all stored using the same method and for a similar period of time (see Crawley et al., 2016). For this reason, we believe that for the purpose of this analysis, the storage method does not constitute an important confounding factor.

Finally, as in every scat survey, morphological identification of samples in the field is not completely reliable (Davison et al., 2002) and it is possible that some samples we assigned to red foxes were from domestic dogs. This is a confounding factor we are aware of, but we deemed it relatively uninfluential in the overall analysis. This is mainly due to dietary differences between dogs and foxes, particularly in urban areas: Domestic dogs tend to have a anthropogenic diet that results in scats without hair or bone fragments in them (Heinemeyer et al., 2008), which makes identification of fox scats relatively reliable. Of course, the use of genetic identification techniques would have eliminated the uncertainty completely, but it would also have greatly reduced our sample size since this kind of analysis tends to have a relatively high level of failure on fecal samples (de Groot et al., 2015; Rutledge et al., 2009), particularly if the sample is not fresh (>24 hr old).

Our study highlights the complexity of the interaction between the urban environment, the wildlife hosts that live in it, and their GI parasite community. Our results showed how measuring socio‐economic and ecological variables at a very fine‐scale within an urban environment helped identify which variables may be affecting both the marking behavior of foxes and driving higher GI parasite diversity and prevalence. While our results are only applicable to the city of Edinburgh, the methodology we developed for this study has the potential to be generalized to a much greater degree, allowing for more meaningful comparison between different urban areas which present different characteristics. Importantly, we show that not all urban environments are the same and that including fine‐scale landscape characteristics in these kinds of studies is a vital step toward a better understanding of the underlying mechanisms driving infection dynamics in urban environments.

## CONFLICT OF INTEREST

None declared.

## AUTHOR CONTRIBUTIONS

**Lisa V. Gecchele:** Conceptualization (equal); data curation (lead); formal analysis (lead); investigation (lead); methodology (lead); validation (lead); visualization (lead); writing – original draft (lead); writing – review and editing (lead). **Amy B. Pedersen:** Data curation (supporting); formal analysis (supporting); investigation (supporting); methodology (supporting); supervision (supporting); validation (supporting); writing – review and editing (supporting). **Matthew Bell:** Data curation (supporting); formal analysis (supporting); investigation (supporting); methodology (supporting); supervision (lead); validation (supporting); writing – review and editing (supporting).

### Open Research Badges

This article has been awarded Open Materials, Open Data, Preregistered Research Designs Badges. All materials and data are publicly accessible via the Open Science Framework at https://doi.org/10.5061/dryad.wwpzgmsgk.

## Supporting information

Appendix S1

## Data Availability

The data presented in this study and the code used to conduct the analysis are available in Dryad (https://doi.org/10.5061/dryad.wwpzgmsgk).
