# Supplementary material for: Fine‐scale variation within urban landscapes affects marking patterns and gastrointestinal parasite diversity in red foxes
Source: Ecol Evol. 2020 Nov 19;10(24):13796–809. doi: 10.1002/ece3.6970 (PMC7771116; doi:10.1002/ece3.6970)
Supplement: Supplementary file 1 — Appendix S1 [file ECE3-10-13796-s001.docx]

**Appendix S1**

Fine-scale variation within urban landscapes impacts marking patterns and gastrointestinal parasite diversity in red foxes

Lisa V Gecchele*, Amy B Pedersen and Matthew Bell

Institute of Evolutionary Biology, School of Biological Sciences, University of Edinburgh, Kings Buildings, Ashworth Laboratories, Edinburgh EH9 3FL UK

*Correspondence email: lisa.gecchele@gmail.com

Methods

Detecting spatial autocorrelation
The spatial autocorrelation level in the model was assessed by constructing a variogram of the Pearson’s residuals of the model. Variograms represent the variability between datapoints at various (increasing) distances; if the points are distributed along a flat line, this indicates that no autocorrelation occurs. If the variability is lower at shorter distances (close datapoints are more similar on average), then autocorrelation is present in the dataset and should be accounted for in the model. In our case the variogram shows a clear autocorrelation structure to about the 1.5Km distance, after which the semivariance levels off.


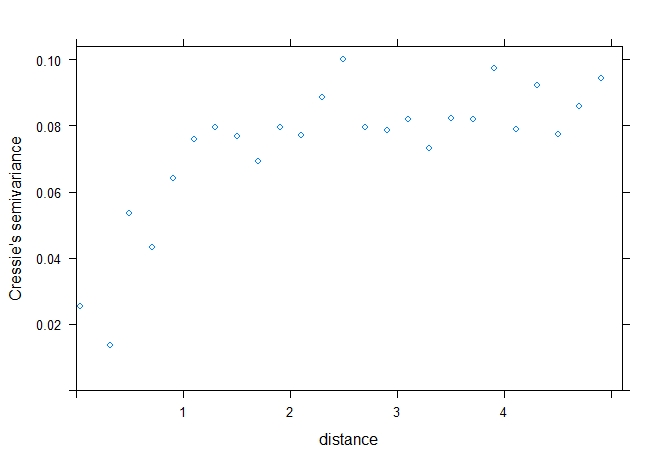


Figure S1 – Variograms regarding scat distribution model. The distance in the X axis is expressed in kilometres.

Results

Results of the single species models

Single species models were run to evaluate the effect of socio-economic and ecological variables on the infection risk (presence/absence; Table S1) and egg/oocyst burden (Table S2) of each parasite taxa identified form red fox faecal samples collected across green spaces sites throughout the urban landscape of Edinburgh UK. The models fit to *Taenia spp* nor the *T. canis* burden model did not data converge, likely due to the low prevalence of each parasites in our dataset, see Table 2 in the main text.

|  | Euculeus Aerophilus | | | | Uncinaria stenocephala | | | Toxocara canis | | | Coccidia | | | | |
| --- | --- | --- | --- | --- | --- | --- | --- | --- | --- | --- | --- | --- | --- | --- | --- |
| Fixed effects | Estimate | SE | | t-value | Estimate | SE | t-value | Estimate | SE | t-value | Estimate | | SE | | t-value |
| Intercept | 4.821 | 3.264 | | 1.477 | -1.177 | 1.211 | -0.971 | -24.356 | 8139.40 | -0.003 | 1.384 | 3.452 | | | 0.401 |
| Road Cover | 0.106 | 0.213 | | 0.497 | 0.195 | 0.239 | 0.816 | 0.523 | 0.476 | 1.100 | 0.249 | 0.221 | | | 1.124 |
| Traffic counts | 0.236 | 0.171 | | 1.373 | 0.249 | 0.183 | 1.363 | 0.403 | 0.281 | 1.434 | -0.079 | 0.205 | | | -0.384 |
| Population Density | 0.284 | 0.180 | | 1.577 | -0.127 | 0.195 | -0.652 | -0.294 | 0.354 | -0.830 | 0.072 | 0.215 | | | 0.335 |
| Green Space Ratio | 2.347 | 1.475 | | 1.591 | **3.442** | **1.689** | **2.037** | 4.536 | 3.417 | 1.327 | 0.422 | 0.260 | | | 1.622 |
| Green Space Variability | 0.358 | 0.207 | | 1.728 | 0.233 | 0.226 | 1.029 | 0.592 | 0.420 | 1.408 | -0.076 | 0.216 | | | -0.355 |
| Sampling period (Autumn) | **-1.069** | **0.334** | | **-3.199** | 0.083 | 0.334 | 0.250 | 0.134 | 0.530 | 0.253 | 0.083 | 0.261 | | | 0.317 |
| Site Area (Log) | -0.939 | 0.679 | | -1.382 | -0.843 | 0.739 | -1.141 | 18.618 | 8139.39 | 0.002 | 0.998 | 0.7107 | | | 1.405 |
| Urban Wilderness (1) | **-1.616** | **0.740** | | **-2.182** | 0.271 | 0.786 | 0.345 | 19.431 | 8139.39 | 0.002 | **1.835** | **0.799** | | | **2.296** |
| Urban Wilderness (2-3) | -0.554 | 0.411 | | -1.345 | -0.169 | 0.240 | -0.708 | 0.115 | 0.783 | 0.148 | -0.358 | 0.433 | | | -0.826 |
| Managed Vegetation level (1-2) | -0.074 | 0.654 | | -0.113 | -1.174 | 0.749 | -1.567 | -1.055 | 1.466 | -0.720 | 0.0503 | 0.693 | | | 0.073 |
| Managed Vegetation level (3-4) | -1.058 | 0.586 | | -1.805 | -1.095 | 0.637 | -1.717 | -0.864 | 1.165 | -0.742 | -0.373 | 0.595 | | | -0.628 |
| Random effects | Variance | |  | | Variance |  | | Variance |  | | Variance | | |  |  |
| *Site ID* | 5.22 e^-09^ |  | |  | 5.15 e^-09^ |  |  | 0.6614 |  |  | 5.22 e^-09^ |  | | |  |
| *Spatial effect* | 0.0556 |  | |  | 0.2507 |  |  | 6.64 e^-10^ |  |  | 0.0556 |  | | |  |

Table S1 – The GLMM model output for the single-species parasite prevalence (presence/absence) regarding E. aerophilus, U. stenocephala, T. canis and coccidian parasites. The variables included in the models are listed on the left, along with estimate, SE and t-values. The variance explained by the random effects are also included at the bottom. Significant terms are highlighted in bold (t-values higher than 2 are considered significant). The results regarding the Taenia spp data are not included since the model did not converge.

|  | *Euculeus Aerophilus* | | | | *Uncinaria stenocephala* | | | Coccidia | | |
| --- | --- | --- | --- | --- | --- | --- | --- | --- | --- | --- |
| Fixed effects | Estimate | SE | | t-value | Estimate | SE | t-value | Estimate | SE | t-value |
| *Intercept* | 3.626 | 2.460 | | 1.473 | -2.298 | 1.376 | -1.670 | 2.291 | 5.894 | 0.388 |
| *Road Cover* | 0.264 | 0.154 | | 1.715 | 0.443 | 0.287 | 1.542 | 0.204 | 0.380 | 0.537 |
| *Traffic counts* | 0.117 | 0.124 | | 0.945 | 0.523 | 0.205 | 2.545 | -0.023 | 0.329 | -0.070 |
| *Population Density* | 0.220 | 0.146 | | 1.505 | -0.065 | 0.214 | -0.305 | -0.119 | 0.303 | -0.394 |
| *Green Space Ratio* | 1.691 | 1.094 | | 1.545 | **4.607** | **2.058** | **2.238** | 0.692 | 0.473 | 1.463 |
| *Green Space Variability* | 0.288 | 0.157 | | 1.828 | 0.315 | 0.276 | 1.144 | -0.154 | 0.409 | -0.376 |
| *Sampling period (Autumn)* | **-1.146** | **0.274** | | **-4.177** | 0.339 | 0.401 | 0.843 | -0.339 | 0.582 | -0.581 |
| *Site Area (Log)* | -0.179 | 0.517 | | -0.347 | -1.018 | 0.771 | -1.320 | 1.833 | 1.447 | 1.266 |
| *Urban Wilderness (1)* | **-1.333** | **0.597** | | **-2.231** | 0.434 | 0.829 | 0.524 | 2.493 | 1.534 | 1.624 |
| *Urban Wilderness (2-3)* | -0.519 | 0.314 | | -1.655 | -0.214 | 0.278 | -0.772 | -0.680 | 0.725 | -0.937 |
| *Managed Vegetation level (1-2)* | 0.204 | 0.491 | | 0.416 | **-1.893** | **0.920** | **-2.057** | -0.115 | 1.300 | -0.089 |
| *Managed Vegetation level (3-4)* | -0.744 | 0.427 | | -1.739 | -1.322 | 0.746 | -1.772 | -0.892 | 1.041 | -0.856 |
| Random effects | Variance | |  | | Variance |  | | Variance |  | |
| *Site ID* | 5.93 e^-09^ |  | |  | 4.51 e^-09^ |  |  | 4.84 e^-09^ |  |  |
| *Sample* | 1.111 |  | |  | 4.488 |  |  | 9.262 |  |  |
| *Spatial effect* | 3.59 e^-07^ |  | |  | 2.68 e^-09^ |  |  | 1.42 e^-05^ |  |  |

Table S2 – The GLMM model output for the single-species parasite burden (eggs/oocyst per gram) regarding E. aerophilus, U. stenocephala and coccidian parasites. The variables included in the models are listed on the left, along with estimate, SE and t-values. The variance explained by the random effects are also included at the bottom. Significant terms are highlighted in bold (t-values higher than 2 are considered significant). The results regarding the T. canis and Taenia spp data are not included since the models did not converge.
